# Study of Acrosome Formation, Interspecific and Intraspecific, in the Testicular Lobes of Some Pentatomid Species

**DOI:** 10.1673/031.010.13201

**Published:** 2010-08-12

**Authors:** Hederson V. Souza, Mary M. Itoyama

**Affiliations:** UNESP - Universidade Estadual Paulista, Instituto de Biociências, Letras e Ciências Exatas, Departamento de Biologia, Laboratório de Citogenética e Molecular de Insetos (LACIMI), Rua Cristóvão Colombo, 2265, Jardim Nazareth, CEP: 15054-000, São José do Rio Preto, SP

**Keywords:** Heteroptera, Pentatomidae, spermiogenesis, harlequin testicular lobe

## Abstract

The objective was to compare the formation of the acrosome in the testicular lobes of the species *Antiteuchus tripterus* F. and *Platycarenus umbractulatus* F., belonging to the subfamily Discocephalinae, and *Euschistus heros* F., *Mormidea quinqueluteum* L., *Oebalus* sp. and *Thyanta perditor* F., belonging to the Pentatominae (Heteroptera: Pentatomidae). It was found that, in general, the behavior of periodic acid Schiff-positive granules for all of the species analyzed is similar for these species. In the beginning of spermiogenesis, there is a central granule that migrates to one of the extremities of the spermatid, and later, it becomes elongated and cannot be distinguished in the spermatozoa. Some species such as *A. tripterus, E. heros* and *P. umbractulatus* showed significant differences in the behavior of the PAS-positive granule in certain lobes, suggesting the formation of spermatozoa with non-fertile functions.

## Introduction

The testes of Heteroptera are formed by variable numbers of lobes; *Antiteuchus tripterus* F. (Pentatomidae, Discocephalinae), for example, possesses testes constituted by six elongated testicular lobes, with the sixth being internal to the fifth (Souza et al. 2007). *Mormidea quinqueluteum* L., *Oebalus poecilus* Dallas, and *O. ypsilongriseus* De Geer, (Pentatomidae, Pentatominae), analyzed by Souza et al. (2008a), possess three, four and four testicular lobes, respectively. The species *Euschistus heros* F. (Pentatomidae, Pentatominae), *Platycarenus umbractulatus* F. (Pentatomidae, Discocephalinae) and *Thyanta perditor* F. (Pentatomidae, Pentatominae) have six, seven and three elongated lobes, respectively (Souza 2009). Only *A. tripterus*, in these mentioned species possess a *harlequin* lobe, where the species *Platycarenus notulatus* (Pentatomidae, Discocephalinae) is considered an exception in the subfamily Discocephalinae with the *harlequin* lobe being absent (Rebagliati et al. 2005).

The *harlequin* testicular lobe, according to the revision of Rebagliati et al. (2005), differs from other lobes by showing irregular meiotic pairing, non-specific association of the autosomes, anomalous arrangement of the chromosomes in the metaphasic plate, irregular chromosome segregation and cell fusion, consequently resulting in the production of spermatozoa with highly variable chromosome number. In the literature, there are records of 23 species belonging to 15 genera of three subfamilies (Discocephalinae, Edessinae and Pentatominae) that possess this lobe (Rebagliati et al. 2005; Souza et al. 2007).

An essential structure of spermatozoa for the recognition and penetration of the ovum, inducing fertilization, is the acrosome that is formed by the Golgi apparatus (Phillips 1970; Baccetti 1972). The development of the acrosome begins with a spherical body, the pre-acrosomal granule. This structure results from the fusion of vesicles produced by the Golgi apparatus, and it is gradually modified until reaching its final form. The size, forms and internal structure of the mature acrosome vary among the different species of animals (Anderson and Personne 1975). More exact and detailed investigations in the Pentatomidae were carried out by Bowen (1922) who analyzed the bodies formed by the Golgi apparatus and the dictyosomes of the spermatocytes to the spermatozoa, and he showed that they are intimately involved in the formation process of the acrosome (Bowen 1922, 1924).

Using the periodic acid Schiff (PAS) technique, Schrader and Leuchtenberger (1951) analyzed the structure of the acrosome in *Arvelius albopunctatus*, a species of the Pentatomidae that contains the *harlequin* lobe, and they demonstrated that the Schiff's reagent reacts with 1,2 glycol groups in polysaccharides. Thus, the acrosome, besides structures including the Golgi apparatus and dictyosomes, forms the acroblast, and they show a positive reaction with this stain. This study allowed the demonstration that *A. albopunctatus* has six lobes, where the third and fifth contain large cells, the fourth smaller cells, and the first, second and sixth lobes normal-sized cells. Still, they found that the spermatozoa possess different acrosome sizes when comparing their lobes. These authors also emphasized that spermatozoa with very large acrosomes could show impaired fertilization (Schrader and Leuchtenberger 1951).

Thus, the objective of the present work was to compare acrosome formation, interspecific and intraspecific, in species belonging to the subfamilies Pentatominae (*E. heros, M. quinqueluteum, Oebalus* sp. and *T. perditor*) and Discocephalinae (*A. tripterus, P. umbractulatus*), using PAS staining.

## Methods and Materials

Fifteen adult males of each species were collected in São José do Rio Preto (20°47′32″ S, 49°21′37″ W), SP, Brazil, and their testes were removed. *P. umbractulatus* possessed seven lobes, *Antiteuchus tripterus* had six testicular lobes, the fifth being the *harlequin* lobe that was internal to the sixth lobe, *E. heros* had six testicular lobes, *Oebalus* sp. four lobes and *M. quinqueluteum* and *T. perditor* three lobes.

These testicular lobes were separated and fixed in Carnoy's (ethanol:acetic acid, 3:1). The slide containing the material was dipped in periodic acid for 15 min and then stained with Schiff's reagent (Garcia 1990) to determine the presence of polysaccharides in the cells. The microphotographs of cells in spermiogenesis, i.e., the final stage of spermatogenesis that leads to the maturation of spermatids into mature, motile spermatozoa, were captured with a AXIOSKOP 2 ZEISS light microscope (AXIO VISION program) of the Laboratory of Morphology in the Department of Biology, IBILCE / UNESP, São José do Rio Preto- SP.

## Results

**Figure 1.**
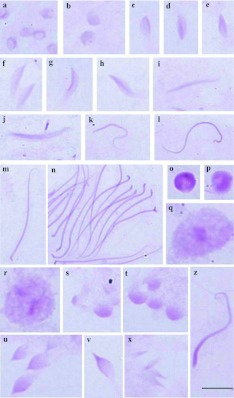
Cells of the testicular lobes of *Antiteuchus tripterus* stained with PAS. **a–n** - Cells of lobes 1–3; **a, b** - round spermatid evidencing PAS-positive granules dispersed throughout the cell; (**c–h**) spermatid in elongation with PAS-positive reaction located in the periphery of the cell (**c–g**) or in the center (**h**); **i–n** - spermatids in the final stage of development without PAS-positive reaction; **o, p** - round spermatids of lobe 4 with intense PAS-positive reaction; **q, r** round spermatid of lobe 6 with PAS-positive reaction in the center; **s–z** - cells of lobe 5; **s, t** - round spermatid: note the different sizes of the cells and PAS-positive reaction; **u–x** - large spermatids (**u**), medium (**v**) and small (**x**); **z**- spermatid in the final stage of development. Bar= 10 *µ*m. High quality figures are available online.

**Figure 2.**
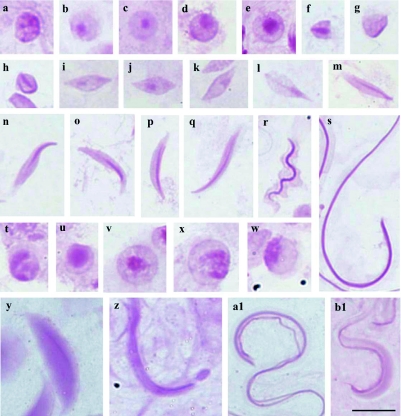
Cells of the testicular lobes of *Euschistus heros* stained with PAS. **a–s** - Cells of lobes 1–3; **a–e** - round spermatid evidencing in (**a**) several dispersed PAS-positive granules in the cell or with the PAS-positive reaction in the center of the cell (**c, e**); **h–q** - medium spermatids with PAS-positive granules moved to one of the extremities of the cell (**h–k**) and later they are elongated towards the center (**l–q**); **r, s** - spermatids in the final stage of development evidencing PAS-positive reaction in whole cell; **t, u** - round spermatids of lobe 5 evidencing strong PAS-positive reaction; **v–y** - cells of lobes 4 and 6 showing in (**v–z**) round spermatids with intense PAS-positive reaction moved to periphery; **w, y** - spermatids in the final stage of development with PAS- positive reaction in the inferior head region; **a1, b1** - spermatid in the final stage of development of lobe 5 with a wide PAS-positive reaction throughout the cell. Bar= 10 *µ*m. High quality figures are available online.

**Figure 3.**
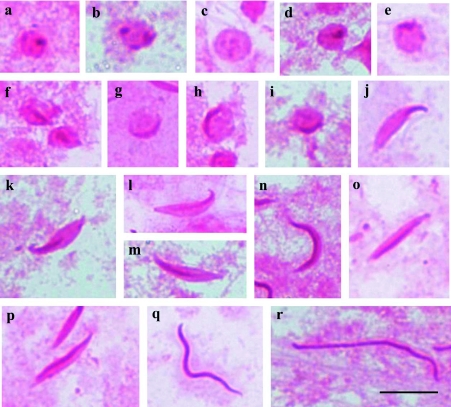
Cells of the testicular lobes of *Mormidea quinqueluteum* stained with PAS. **a–i** - Round spermatids evidencing in (**a, b**) several dispersed PAS-positive granules in the cell, in (**c, d**) the PAS-positive reaction is located in the center of the cell and moved to the periphery (**e, f**), acquiring the C shape (**g–i**); **j–p** - medium spermatids with PAS- positive granule at one of the extremities (**j, k**), which is elongated toward the cell center (**m**) or is at the periphery of the cell (**n–p**); **q, r** spermatids at the end of elongation evidencing a continuous PAS-positive reaction in whole head. Bar= 10 *µ*/m. High quality figures are available online.

**Figure 4.**
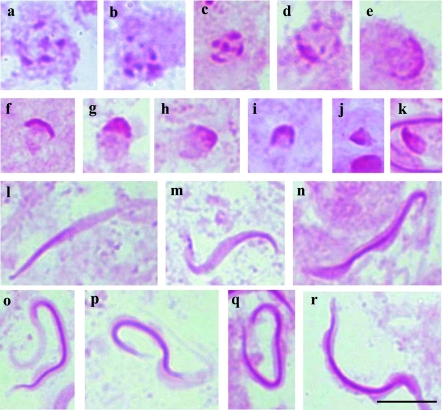
Cells of the testicular lobes of *Oebalus* sp. stained with PAS. **a–f** - Round spermatids with several dispersed PAS-positive granules in the cell (**a, c**), moved to the periphery (**d, f**); **g–k** - spermatids in the beginning of development with PAS-positive reaction at one of the extremities of the cell; **l–n** - medium spermatids with PAS-positive granule being elongated towards other extremity of the cell; **o–r** - spermatids in the final stage of development acquiring the C shape (**g–i**); **j–p** - medium spermatids evidencing a continuous PAS-positive reaction in whole head. Bar= 10 *µ*m. High quality figures are available online.

**Figure 5.**
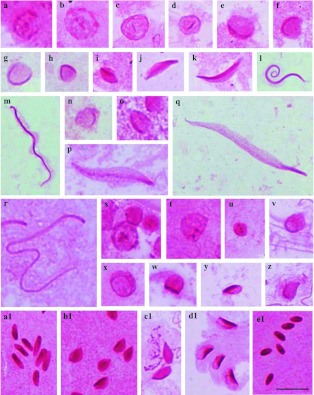
Cells of the testicular lobes *of Platycarenus umbractulatus* stained with PAS. **a– m** - Cells of lobes 1–3 and 7; **a–g** round spermatids evidencing in (**a, b**) several dispersed PAS-positive granules in the cell, in (**c, d**) the PAS-positive reaction is located in the center of the cell, moved to the periphery forming a C shape (**e–g**); **h–k** - medium spermatids with PASpositive reaction on one on the sides; **l–m** - spermatids in the final stage of development with PAS-positive reaction in whole head, **n–r** - Cells of lobes 4 and 6; **n, o** - spermatids in elongation with PAS-positive reaction forming a C shape, **p, q** medium spermatids with PAS-positive granules throughout the cell, more evident at one of the extremities, **r** spermatid in the final stage of development with a PAS- positive reaction at one of the extremities, **s–e1** - Cells of testicular lobe 5; **s–x** round spermatids with different PAS-positive reaction, **z–el** - spermatids in development with different PAS-positive reaction. Bar= 10 *µ*m. High quality figures are available online.

**Figure 6.**
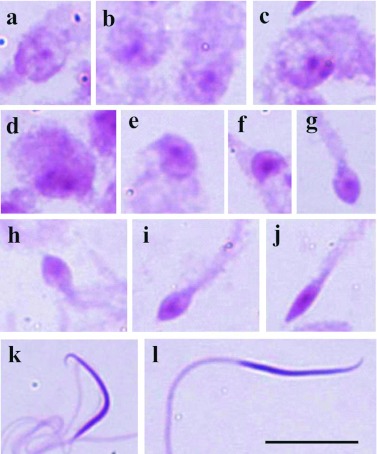
Cells of the testicular lobes of *Thyanta perditor* stained with PAS. **a–f** - Round spermatids evidencing PAS-positive reaction located in the center of the cell; **g–i** - the PAS-positive reaction remains in the center of the cell until the final stage of elongation of the spermatid; **j** - elongated spermatid demonstrating a notable elongation of the central PAS-positive reaction; **k, l** - spermatids in the final stage of development with continuous PAS-positive reaction in the whole head. Bar= 10 *µ*m. High quality figures are available online.

All species analyzed (*A. tripterus, E. heros, M. quinqueluteum* L., *Oebalus* sp., *P. umbractulatus* and *T. perditor*) in general, had the beginning of the formation of the PAS-positive granules in the beginning of spermiogenesis (spermatocytes or round spermatids). The presence of several dispersed granules can be observed throughout the cell (Figures 1a, b; 2a; 3a, b; 4a–c; 5a, b), where these join forming a PAS-positive reaction located only in the center (Figures 2c–e; 3c, d; 5c, d; 6a–f) which moves to one of the extremities of the cell (Figures 2f–k; 3e–i; 4d–i; 5e–h), and in some cases, acquiring a C shape (Figures 3g–i; 4f; 5g, h).

In the medium spermatid, there is elongation of the PAS-positive reaction (Figures 1c–j; 2l–q; 3j–p; 4j–n; 5i–k). However, in some cases, it is observed that the cell stays approximately the same length (Figures 1f–j; 2n–q; 3j–p 4l, m; 5j, k). At the end of the development of the spermatid, the PAS-positive reaction already reaches the whole length of the spermatid (Figures 1k–m; 2r, s; 3q, r; 4o–r; 5l, m; 6k, 1).

The PAS-positive reaction obtained in the species *A. tripterus* was weak compared to other species. However, some differences can be observed in lobes 4, 5 and 6. In lobe 4, the round spermatids show intense PAS-positive reaction in the cell (Figure 1o, p); the round spermatids of lobe 6 show a strong PAS-positive granule in the center (Figure 1q, r). In lobe 5, the PAS-positive reaction is proportional to the size of the spermatid (Figure 1s–z).

The species *E. heros* showed in lobes 4, 5 and 6 a larger and very evident PAS-positive granule, but with a behavior similar to that of other lobes (Figure 2t–z). PAS-positive granule was verified in the posterior region in final stage of spermatid elongation in lobes 4 and 6 (Fig 2z) and in lobe 5; the spermatids in the final stage of elongation displayed a large PAS-positive region along the head (Figure 2a1, b1).

In the species *P. umbractulatus*, lobes 1–3 and 7 showed a behavior similar to that described previously for *E. heros*; however, a marked PAS-positive reaction at one of the extremities and a less pronounced one throughout the spermatid was observed in lobes 4 and 6, in the beginning of the spermatid elongation (Figure 5p). During spermatid development, the longitudinal PAS-positive reaction became less evident, where the PAS-positive granule remained located at the extremity of the cell (Figure 5q, r). In lobe 5, the PAS reaction, in the round spermatid, showed various staining intensities including no apparent reaction (Figure 5s–w). In the beginning of the development of the spermatids, PAS reaction was apparent at one of the extremities (Figure 5z, d1), on one side of the cell (Figure 5y), without apparent reaction (Figure 5a1–c1) or with PAS reaction in the whole cell (Figure 5e1).

The spermatids of *T. perditor* showed a PAS-positive granule located in the center of the cell (Figure 6a–f) which remained until the final stage of spermatid development (Figure 6g–i), where it is possible to show a little elongation in the PAS-positive granule (Figure 6j).

## Discussion

The acrosomal bodies are deposited by the acroblast (Golgi apparatus) near the nuclear envelope, usually before the elongation of the nucleus. If this material is not originally located in the anterior region of the nucleus, it usually migrates toward this region, and eventually moves forward to the terminal region in the mature spermatozoon (Bowen 1924). Bowen (1924) proposed that the relative position of the acrosome could be located anywhere in the periphery of the nucleus, and that it shows, however, an almost universal tendency for some portions of this structure to reach the anterior region of spermatozoa.

In the present work, a similar behavior was observed in acrosome formation for all species analyzed; that is, several PAS-positive granules join into one and move to one region of the cell. It can be verified that there is an elongation of the PAS-positive region during the medium spermatid stage, which is not accompanied by the elongation of the spermatid, as occurs in *E. heros*. This structure could indicate stages of the development of the acrosome during this phase of development of the spermatid, and still, it could indicate that the spermatid elongation does not depend on the elongation of the acrosome in this species. In the final stage of elongation, its detection is no longer possible, since there is no distinction between the nucleus and the PAS-positive region.

Schrader and Leuchtenberger (1951) demonstrated that the synthesis of acrosomal material can exhibit a certain degree of independence from the synthesis of other cellular structures. The physiologic changes in the third and fifth testicular lobes of *A. albopunctatus* induce a great increase in nucleoplasm, cytoplasm and nucleolus, even inducing an increase in acrosomal material. The result is an enormous acrosome that possibly affects the efficiency of the spermatozoon in fertilization. Similarly, investigators have suggested that very discrete alterations in the physiologic milieu could affect acrosome formation, and consequently, they can cause some degree of sterility.

In the species *E. heros*, Souza (2009) found the formation of different sizes in lobes 4 and 6 and the formation of spermatids with atypical morphologic patterns in lobe 5, which would probably be related to non-fertile functions. In our work, it was found that *E. heros* has a larger deposition of PAS-positive granules in lobes 4, 5 and 6 when compared with the other lobes, and that in the final stage of spermatid development, in lobes 4 and 6 there is a strong PAS-positive reaction in the posterior region of the nucleus. As the acrosome is considered a structure related to fertilization, an exaggerated increase in the size of this structure can indicate the substitution of function of the acrosome for the spermatozoa of certain lobes in the same individual, supporting the hypothesis proposed by Souza (2009).

The species *P. notulatus* is considered in literature as an exception, belonging to the subfamily Discocephalinae but not showing a *harlequin* lobe (Rebagliati et al. 2005). In our study, *P. umbractulatus* showed differentiated patterns in some lobes, mainly for lobe 5. This change could be due to different physiologies in the testicular lobes. Lobes 1–3 and 7 revealed similar behaviors, as well as lobes 4 and 6. Lobe 5 showed spermatids, apparently in the same developmental stage, with different PAS-positive reactivity, suggesting that, besides physiologic changes in the testes influencing the formation of the acrosome, changes in cellular physiology can also alter the structure of the acrosome inside the same lobes.

These changes in the structure of the acrosome can affect in some way the individual's fertility. Souza et al. (2007, 2008b) in analyzing the species *A. tripterus* suggested that the changes observed among the testicular lobes could affect the individual's fertility; however, it can be suggested that other non-fertility functions exist, such as supplying additional nutrients, especially nucleoproteins for the development of the ovule (Schrader 1945, 1960a, b), for the females or for the fertilizing spermatozoa (McLain 1998; Swallow and Wilkinson 2002), thereby maintaining the adaptive value of males with non-fertile spermatozoa.

These intraspecific divergences in the formation of the acrosome could be due to the formation of spermatozoa with differentiated function, as occurs in the species *A. tripterus, P. umbractulatus* and *E. heros*. Similar characteristics occurred interspecifically, as seen when comparing the same lobes in the species *A. tripterus* and *P. umbractulatus* belonging to the subfamily Discocephalinae and *E. heros* belonging to the Pentatominae, which could indicate phylogenetic proximity, while the species *Oebalus* sp., *M. quinqueluteum* and *T. perditor* belonging to the Pentatominae share the same characteristics of acrosome formation, indicating close relationship. However, more investigations are necessary to investigate the function and phylogenetic relation involving the formation of spermatozoa in the Pentatomidae species.

## Abbreviation

PAS: periodic acid Schiff technique
